# Supplementary material for: The causal role of circulating inflammatory markers in osteoporosis: a bidirectional Mendelian randomized study
Source: Front Immunol. 2024 Jul 18;15:1412298. doi: 10.3389/fimmu.2024.1412298 (PMC11291241; doi:10.3389/fimmu.2024.1412298)
Supplement: Supplementary file 1 [file DataSheet_1.zip › supplementary material/CIM-OSTEOPOROSIS /GCST90274838.csv_forest.pdf]

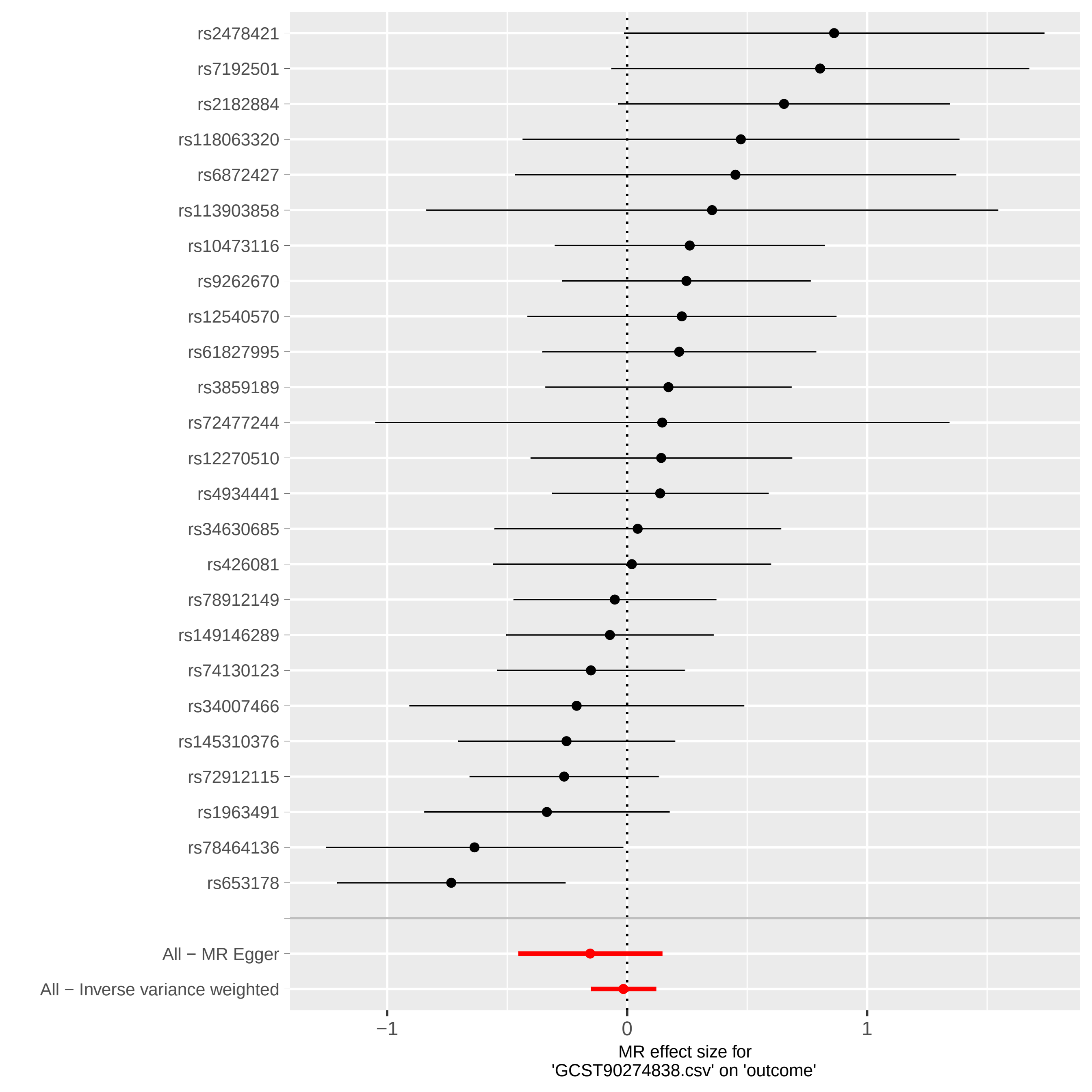
rs2478421
rs7192501
rs2182884
rs118063320
rs6872427
rs113903858
rs10473116
rs9262670
rs12540570
rs61827995
rs3859189
rs72477244
rs12270510
rs4934441
rs34630685
rs426081
rs78912149
rs149146289
rs74130123
rs34007466
rs145310376
rs72912115
rs1963491
rs78464136
rs653178
All − MR Egger
All − Inverse variance weighted
−1
0
1
MR effect size for
'GCST90274838.csv' on 'outcome'
